# Encapsulating peritoneal sclerosis caused by cell-free and concentrated ascites reinfusion therapy: a case report

**DOI:** 10.1186/s13256-021-02679-8

**Published:** 2021-02-06

**Authors:** Hideya Itagaki, Suzuki Katuhiko

**Affiliations:** Department of General Surgery, Honjoudaiichi Hospital, Akita, Iwabuchishita, Yurihonnjou 110015-8567 Japan

**Keywords:** Encapsulating peritoneal sclerosis (EPS), Hemodialysis, Cell-free and concentrated ascites reinfusion therapy (CART), Bowel obstruction, Cirrhosis

## Abstract

**Background:**

Encapsulating peritoneal sclerosis (EPS) is a rare condition in which the small intestine is covered by an inflammatory fibrocollagenous membrane; the exact etiology of EPS is unclear. Herein, we report the case of our patient who underwent hemodialysis and cell-free and concentrated ascites reinfusion therapy (CART) and was diagnosed with EPS.

**Case presentation:**

A 64-year-old Japanese man visited our emergency department with a chief complaint of abdominal pain. He had a medical history of cirrhosis due to hepatitis C for 25 years. He had undergone partial resection of the small intestine 2 years earlier for an incarcerated hernia. One year earlier, he experienced renal failure due to hepatorenal syndrome and started hemodialysis three times a week and CART twice a month. Physical examination of the abdominal wall revealed a lack of peristalsis of the intestinal tract and strong tenderness on palpation.

Because hernia of the small intestine was found on computed tomography, we suspected strangulation ileus, requiring emergency operation. When the abdomen was opened, the entire small intestine was found to be wrapped in a fibrous membrane and constricted by it. The patient was diagnosed with EPS; hence, during surgery, the fibrous membrane was excised, resulting in decompression of the intestinal tract and subsequent recovery.

**Conclusions:**

EPS is thought to be related to various elements, but no case of EPS induced by CART has been reported to date. EPS should be considered in the differential diagnosis of small bowel obstruction in patients undergoing CART for refractory ascites.

## Background

Encapsulating peritoneal sclerosis (EPS) is a rare syndrome in which the small intestine or other intra-abdominal organs become wrapped by fibrous collagen membranes [1], resulting in intestinal obstruction. EPS is divided into idiopathic and secondary forms, and secondary EPS is thought to be related to various elements such as drug use, peritoneal dialysis, and infection [2].

However, no case of EPS induced by cell-free and concentrated ascites infusion therapy (CART) has been reported to date. A patient on hemodialysis who was undergoing CART developed EPS, and we present his case below.

## Case presentation

**Fig. 1:**
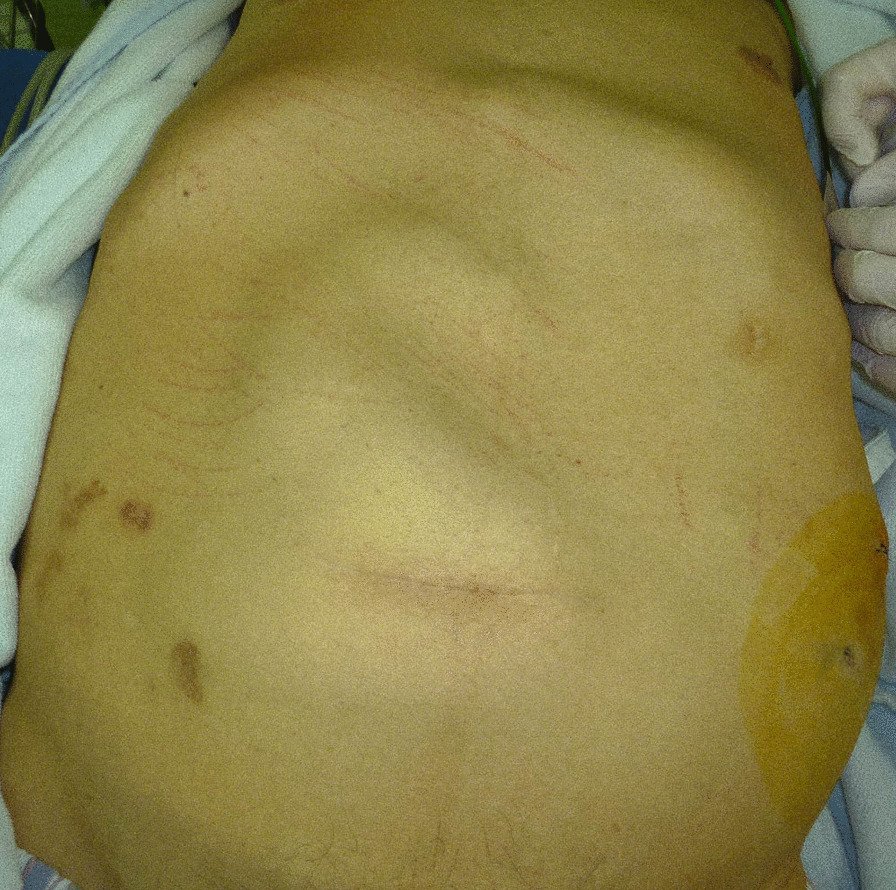
The intestinal tract without peristalsis

**Fig. 2:**
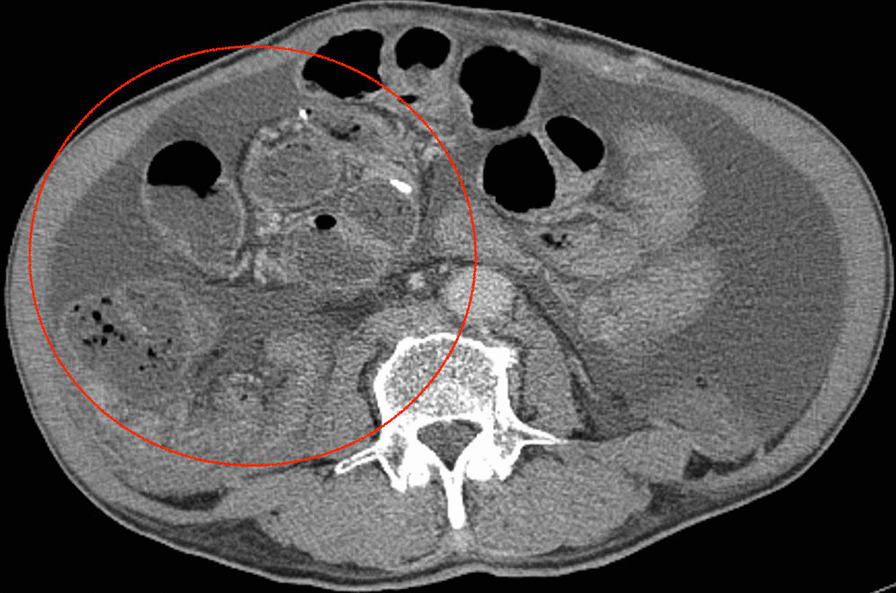
The small intestine and mesenteric blood vessels were aggregated (red circle)

**Fig. 3:**
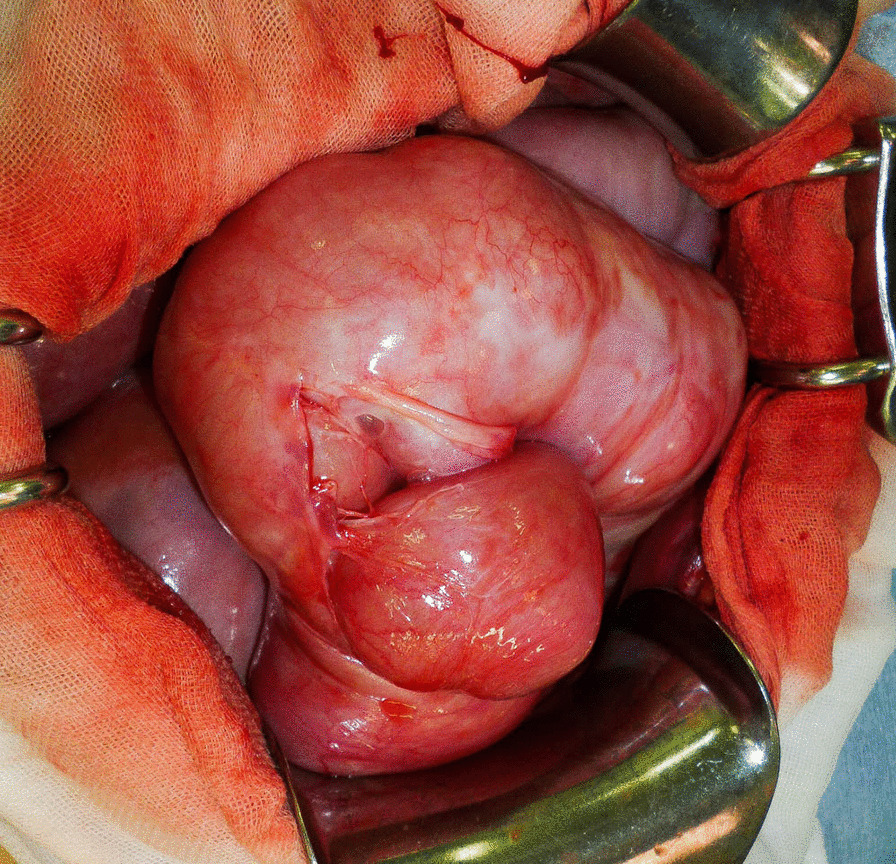
The small intestine was wrapped in a fibrous membrane

A 64-year-old Japanese man visited the emergency department with a chief complaint of abdominal pain. His had a medical history of cirrhosis due to hepatitis C for 25 years. He had undergone partial resection of the small intestine 2 years earlier for an incarcerated hernia. One year earlier, he had experienced renal failure due to hepatorenal syndrome and started hemodialysis three times a week and CART twice a month. He was administered ursodeoxycholic acid (30 mg), alfacalcidol (0.5 μg), methenolone acetate (10 mg), and folic acid (10 mg). He had no significant family history and did not consume alcohol or smoke. On the day of his visit, he received CART in the morning, and after lunch, symptoms of abdominal pain appeared, and the pain gradually worsened; thus, he was admitted to the emergency outpatient clinic. His vital signs at the time of the visit were as follows: blood pressure, 119/74 mmHg; pulse rate, 54 beats per minute; temperature, 37.6 °C. On physical examination of the abdominal wall, we found a lack of peristalsis of the intestinal tract, accompanied by severe oppressive pain (Fig. 1). His blood test results showed anemia (hemoglobin level of 8.3 g/dL), thrombocytopenia (platelet count of 13.0×10^4^/μL), and elevated inflammatory response (C-reactive protein level of 9.02 mg/dL). Renal dysfunction was also noted, with urea nitrogen level of 37.1 mg/dL and creatinine level of 5.58 mg/dL, but because he was post-dialysis, his renal function showed improvement from laboratory values prior to the last dialysis session that he had undergone. His liver enzyme levels were within normal ranges: aspartate transaminase, 21 U/L; alanine transaminase, 14 U/L; and lactate dehydrogenase, 254 U/L. We performed computed tomography (CT) because we suspected strangulated bowel obstruction (SBO), and we performed a puncture to collect ascites fluid for examination. The ascites was not bloody, but was yellow and transparent. However, his CT scans showed aggregation of the small intestine and mesenteric blood vessels (Fig. 2), so we confirmed the diagnosis of SBO due to an internal hernia and planned an emergency surgery. When the abdomen was opened, the entire small intestine was found to be wrapped in a fibrous membrane that constricted the small intestine (Fig. 3). We then suspected EPS and planned to remove the fibrous membrane. However, the fibrous membrane was tightly attached to the serosa of the intestinal tract, and if we completely removed it, the intestine would be severely damaged. After part of the fibrous membrane was removed, the pressure on the intestinal tract decreased, so we performed partial excision of the membrane. After the operation, a nasal tube was inserted, and the tube was not removed until the drainage had decreased. The nasal tube was removed on the third postoperative day, and on the fourth postoperative day, he was able to have meals and was subsequently discharged. Pathological examination of the membrane excised during surgery showed the presence of fibrous connective tissue, consistent with the features of EPS. It has been more than 2 years since the surgery, and there has been no sign of recurrence/another episode of SBO.

## Discussion

Herein, we present the case of a 64-year-old man who visited our emergency outpatient department with a chief complaint of abdominal pain and underwent emergency surgery because of strangulated ileus. He was diagnosed with EPS caused by CART. This case is novel, as it showed that EPS can be caused by CART.

EPS was first reported in 1907 by Owtschinnikow as “peritonitis chronica fibrosa incapsulata.” It was also described as abdominal cocoon syndrome or sclerosing encapsulating peritonitis [3]. The etiology of this rare syndrome, in which the small intestine or other abdominal organs are wrapped by a fibrous collagen membrane, is unknown. Several factors may cause sclerosis, such as cytokines releasing fibrin-like substances that lead to the formation of a fibrillar collagen membrane [4, 5].

EPS is classified into idiopathic (primary) and secondary forms. Adolescent women in tropical and subtropical regions often have idiopathic EPS, usually associated with gynecological diseases. However, other studies have reported that men are more likely to develop the disease than women [6, 7]. Secondary EPS is thought to be triggered by local or systemic factors [4, 5], associated with drug use (beta-blockers/methotrexate), peritoneal dialysis, infections (tuberculosis/cytomegalovirus/peritonitis), autoimmune diseases (systemic lupus erythematosus/sarcoidosis), cirrhosis, gastrointestinal malignancy, abdominal surgery, and trauma [2, 8].

In our patient’s case, he did not take beta-blockers and had no history of autoimmune disease or sarcoidosis, his ascites culture was negative, and he had no infectious diseases. Thus, we identified three possible causes of EPS and resultant persistent serositis: cirrhosis, abdominal surgery, or CART. Cirrhosis seemed to be an unlikely cause, since he had had this disease for 25 years, and there was no evidence of EPS at the time of his surgery for abdominal hernia 2 years before the current visit. Furthermore, abdominal surgery was performed with a small incision in the peritoneum around the umbilicus, and generalized peritonitis was not observed; thus, these operations did not seem to be the cause of persistent serositis. Hence, CART seemed to be the only cause, since his EPS developed after he started CART a year ago. CART is administered to diuretic-resistant patients with ascites, requiring abdominal punctures at multiple locations on both the left and right sides for draining the ascites. We inferred that his persistent serositis was caused by the invasiveness of the punctures; however, since there are no case reports of EPS caused by CART, we think that further accumulation of data is necessary.

When the patient visited our hospital for abdominal pain, we observed a palpable mass with oppressive pain on physical examination. Typically, patients who have ESP visit the hospital for SBO with nausea/vomiting (54%), abdominal fullness (82%), and abdominal pain (86%). In a few cases, a palpable mass can be found [8, 9]. However, there are no discriminative physical or laboratory findings, so diagnosis is difficult, and in many cases the diagnosis is not made until surgery [10].

CT is useful in the diagnosis of EPS. CT findings usually include thickening of the intestinal wall, localized ascites retention, calcification of the peritoneum and the intestinal wall, and small bowel loops concentrated in the center [11, 12]. We performed CT and found extensive aggregation of the small intestine and mesenteric blood vessels, suggesting the presence of EPS. However, we initially diagnosed him with an internal hernia and confirmed the diagnosis of EPS only during surgery because we had not considered EPS before the surgery. Hence, the correct diagnosis of EPS also depends on whether doctors are familiar with EPS [11, 12].

With regard to treatment options, it is necessary to treat the primary disease as well as EPS; however, the gold standard of treatment is surgery. In one report, 29% of the patients had undergone emergency surgery [6]. The operation involves cutting and removing the collagenous membrane to reduce the pressure on the intestinal tract. Intestinal resection is performed only when the tract cannot be preserved [9]. EPS is classified according to the extent of coverage by the fibrous collagenous membrane: type I, the small intestine is partially covered; type II, the small intestine is entirely covered; type III, in addition to the entire small intestine, other organs are covered [13]. Decompression and resection of the entire membrane layer may be difficult when the coverage is extensive. We cut and excised the collagenous membrane; however, since his EPS level was type III, it was difficult to excise the entire layer. Nevertheless, the patient’s prognosis was good. Thus, partial decompression may be sufficient.

## Conclusion

To the best of our knowledge, there is no previous case report of EPS caused by CART. In the future, the number of patients who undergo CART for intractable ascites is expected to increase; thus, EPS should be considered in the differential diagnosis of SBO in patients who receive CART.

## Data Availability

Data sharing is not applicable to this article as no datasets were generated or analyzed during the current study.
